# ChatGPT-5 vs oral medicine experts for rank-based differential diagnosis of oral lesions: a prospective, biopsy-validated comparison

**DOI:** 10.1007/s10266-025-01242-x

**Published:** 2025-11-17

**Authors:** Asmaa Abou-Bakr, Ahmed El Barbary, Fatma E. A. Hassanein

**Affiliations:** 1 Oral Medicine and Periodontology, Faculty of Dentistry, Galala University, Suez, Egypt; 2 Oral Medicine and Periodontology, Faculty of Dentistry, Cairo University, Giza, Egypt; 3 Oral Medicine, Periodontology, and Oral Diagnosis, Faculty of Dentistry, King Salman International University, El-Tor, Egypt

**Keywords:** Artificial intelligence, Large language model, ChatGPT-5, ChatGPT-4o, Differential diagnosis, Oral lesions, Diagnostic accuracy

## Abstract

Accurate differential diagnosis of oral lesions is challenging. Large language models (LLMs) may support clinicians, but expert-validated evidence on ranked differential lists remains limited. This study aimed to compare ChatGPT-5 with ChatGPT-4o and an oral medicine expert for biopsy-confirmed oral lesions. In this prospective, paired accuracy study, 100 biopsy-confirmed cases with standardized vignettes and photographs were independently assessed to produce Top-5 ranked differentials. Accuracy at Top-1, Top-3, and Top-5 was benchmarked against histopathology; subgroup analyses considered lesion type and case difficulty. Agreement with the expert was evaluated using percent agreement, Cohen’s κ, and AC1. Top-1 accuracies were 52% (ChatGPT-5), 59% (ChatGPT-4o), and 79% (expert; Cochran’s Q, *p* < 0.001). At Top-3, accuracies were 72%, 77%, and 88%; at Top-5, 78%, 83%, and 91%. Inflammatory lesions showed significant Top-1 differences favoring the expert, whereas performance converged at broader ranks. Agreement with the expert improved with broader thresholds: ChatGPT-5 AC1 rose from 0.361 (Top-1) to 0.715 (Top-5), and ChatGPT-4o from 0.336 to 0.767, while κ remained in the fair range. ChatGPT-5 generated clinically useful ranked differentials approaching expert performance at Top-3/Top-5 but lagged at Top-1. Lesion type, particularly inflammatory, influenced accuracy, supporting supervised clinical use. Although large language models may assist in narrowing differential diagnoses, their role in oral medicine remains supportive rather than determinative. Human expertise remains indispensable, and integration into clinical workflows should be restricted to supervised settings until future iterations achieve parity with experts.

**Supplementary Information:**

The online version contains supplementary material available at 10.1007/s10266-025-01242-x.

## Introduction

Timely and accurate diagnosis of oral lesions is essential because delayed or incorrect diagnosis can have serious clinical repercussions [1–4]. When malignant oral lesions like oral squamous cell carcinoma (OSCC) are misdiagnosed, treatment delays can significantly lower survival [5]. According to large-scale cohort studies, early-stage OSCC survival rates decrease from over 80% to less than 40% when diagnosis is postponed for more than 2 months, and a time-to-treatment initiation of more than 40–60 days is linked to a significantly higher mortality risk [6, 7]. Even a 4-week delay in diagnosing OSCC increases the risk of death by 15%, according to a 2020 meta-analysis [8]. These results highlight how urgently oral medicine needs trustworthy diagnostic aids.

A fundamental first step in this diagnostic pathway is for the clinician to become aware that there is an alteration in the mucosa [9, 10]. A key cause of undue delay is the failure to identify abnormal tissue in the first place, a challenge for which formal visual inspection regimens and decision rules have a long history of improvement [11, 12]. This specific study, however, is a response to the following challenge: once a lesion is identified, the formation of a valid differential diagnosis is of utmost relevance to guide appropriate management, e.g., biopsy or referral [13]. It is at the subsequent level of clinical reasoning that artificial intelligence (AI) may be able to assist.

AI has emerged in the medical field as a potential solution, capable of handling complex data processing through image recognition, data mining, and deep learning [14]. In dentistry, AI applications have mainly focused on computer vision models, assisting radiologists and maxillofacial surgeons in diagnosing diseases, anticipating prognoses, or developing treatment plans tailored to individual patients [15–17]. More broadly, digitalization of clinical workflows is increasingly recognized as the future, offering streamlined processes, enhanced precision, and more personalized care [18, 19].

However, the unimodal nature of these traditional AI systems is a major drawback. Usually, they are made to process text or images, but not both at once. This is a serious flaw because clinical diagnosis is multimodal by nature, depending on the combination of clinical signs (text/description), historical narratives (text), and visual inspection (images). As a result, although the body of research is expanding, it is still small and shows that AI’s diagnostic precision for oral diseases frequently falls short of traditional expert diagnosis [15, 20].

Large language models (LLMs) like ChatGPT provide a paradigm shift in this area. Advanced LLMs can interpret and synthesize both photographic evidence and textual clinical vignettes within a single model, a capability not found in earlier AI tools. They can mimic a more comprehensive, clinician-like diagnostic approach, thanks to this capability, demonstrating potential in many medical domains for lesion diagnosis and prediction [21, 22].

Nevertheless, in oral medicine, there is still a gap in knowledge regarding the accuracy of the newest frontier models, namely ChatGPT 5 for oral lesion differentials in comparison to expert clinicians, though, as the majority of published dental/oral studies to date have concentrated on non-LLM computer vision models or earlier LLM generations (e.g., GPT-3.5/4, GPT-4o) [15, 16, 23–26]. LLMs can help learners, according to initial dental education and licensing examination analyses, but they also show variability across domains, which emphasizes the significance of domain-specific validation [27, 28].

Crucially, the use of differential diagnoses in actual clinical practice is reflected in rank-based evaluation. By encouraging more focused research, directing the choice of biopsy site, or avoiding premature diagnostic closure, the presence of the correct diagnosis among the top three to five options can still significantly impact decision-making, even if it is not the first option on the list (Top 1). Therefore, evaluating performance at the Top-1, Top-3, and Top-5 levels offers a more clinically meaningful way to assess whether LLMs can serve as high-recall, safe adjuncts in oral medicine.

In light of this, we aimed to assess ChatGPT-5’s diagnostic accuracy in making differential diagnoses for oral lesions and contrast its results with those of seasoned professionals. Through the stratification of results by lesion category and difficulty, as well as the analysis of rank-based recall and agreement, our study fills a timely evidence gap and provides insight into whether next-generation LLMs can serve as high-recall, safe adjuncts in oral medicine decision-making. Our goal is to establish a rigorous, expert-anchored benchmark for clinical translation, building on previous reports of promising LLM results on oral case vignettes and strong AI performance in oral cancer screening.

## Materials and methods

### Study design and aim

This was a prospective, paired comparative diagnostic accuracy study designed to evaluate the ability of **ChatGPT-5** and **ChatGPT-4o** to generate differential diagnosis lists for oral lesions, benchmarked against human oral medicine experts. The study assessed model performance at Top-1, Top-3, and Top-5 diagnostic ranks and explored subgroup accuracy by lesion type and case difficulty. Inter-observer agreement and predictors of diagnostic accuracy were also analyzed.

### Ethical considerations

All participants provided written and oral informed consent in line with the Declaration of Helsinki (1964) and subsequent amendments. Approval was obtained from the Faculty of Dentistry, Ain Shams University (FDAs-Rec ID032209). Patient data and images were anonymized and used exclusively for research and publication purposes, with strict preservation of confidentiality.

### Sample size calculation

The minimum number of cases required to compare the diagnostic accuracy between **ChatGPT-5** and **ChatGPT-4** in a paired design was calculated using McNemar’s test for matched binary outcomes. Each clinical case was evaluated by both models, enabling within-subject comparison. Estimates of diagnostic accuracy were based on **Tomo et al. (2024) **[29], who reported 64.86% accuracy for ChatGPT-3 and 80.18% for ChatGPT-4 in detecting oral and maxillofacial lesions [29]. With a two-sided significance level of 5% (*α* = 0.05), 80% power (*β* = 0.20), and an anticipated discordance rate of 10–15% between model predictions, the required sample was determined to lie between 88 and 100 cases. As the maximum feasible sample size was set at 100, this number was adopted as the final target, providing sufficient power to detect moderate discordant differences in diagnostic accuracy between the two models. Calculations were performed using Stata 18.0 with the power paired-proportion function, suitable for testing differences in paired binary responses.

### Study setting and participants

Cases were prospectively recruited from partner oral medicine clinics (Ain Shams University, King Salman International University, and Galala University). Adult patients (≥ 18 years) with clinically evident oral lesions requiring diagnostic evaluation were included. Exclusion criteria were prior lesion treatment (surgery/radiotherapy), incomplete clinical data, or poor-quality photographs.

### Case development and reference standard

High-quality intraoral photographs and clinical data (lesion site, dimensions, history, symptoms, and risk factors) were collected according to standardized protocols. Before inclusion, each case was reviewed independently by two board-certified oral medicine specialists (> 10 years’ experience each) to ensure clinical representativeness and adequate documentation. To avoid ambiguity, only cases with complete records and biopsy confirmation were retained, while cases with incomplete data or poor-quality photographs were excluded. This process ensured that both AI models and the expert evaluated biopsy-validated lesions with sufficient clarity for consistent interpretation, without restricting the dataset to only low-complexity cases. Each lesion underwent biopsy, and histopathological evaluation by board-certified pathologists blinded to AI and expert diagnoses served as the reference standard.

### Model input and prompting

Case vignettes were formatted consistently and included structured text plus photographs. To establish task context, both AI models were primed with standardized anchor vignettes (see Supplementary Material, Section S1). After priming, each clinical vignette was presented using the following standardized prompt:

*“According to the clinical case and corresponding photographs in the attachment document, what are the five most likely diagnoses in order of likelihood?”*

To ensure independence between cases, conversation history was cleared before each new vignette.

### Expert comparator

Two board-certified oral medicine experts with more than 10 years of experience independently generated ranked Top-5 differential diagnosis lists for all cases. Discrepancies were resolved by consensus discussion, and the consensus list served as the expert comparator for all analyses.

### Outcomes

#### Primary outcome

- Diagnostic accuracy of **ChatGPT-5**, **ChatGPT-4o**, and the expert, defined as the proportion of correct diagnoses at Top-1, Top-3, and Top-5 ranks compared with the histopathological gold standard.

#### Secondary outcomes

- Subgroup accuracy was stratified by lesion type (inflammatory, benign, reactive, malignant). Lesion type was assigned by independent pathologists based on histopathological diagnosis and disease etiology, to allow subgroup analysis.
- Case difficulty (low, middle, high) was stratified a priori by two board-certified oral medicine specialists (> 10 years’ experience) before AI evaluation. Difficulty was defined based on clinical complexity across all lesion categories: (i) **low**: typical, well-demarcated, common lesions with pathognomonic features and minimal overlap; (ii) **middle**: moderately atypical lesions with overlapping features or diagnostic ambiguity requiring history integration; and (iii) **high**: rare, atypical, or ambiguous lesions with significant overlap or confounding factors. Discrepancies were resolved by consensus. These criteria were adapted from standard oral medicine and pathology references [30, 31] and a previously published framework on diagnostic ambiguity [25].
- Agreement between AI models and the expert, assessed by percent agreement, Cohen’s κ, and AC1.
- Predictors of diagnostic performance, evaluated through multivariable logistic regression.

### Statistical analysis

Diagnostic performance at Top-1, Top-3, and Top-5 ranks was summarized descriptively. Pairwise model–expert comparisons used exact McNemar’s tests with Holm correction, and global differences were assessed via Cochran’s Q. Subgroup analyses stratified cases by lesion type and difficulty. Agreement with the expert was quantified using percent agreement, Cohen’s κ, and AC1. Multivariable logistic regression (per rank) examined lesion type and difficulty as predictors of accuracy, reporting odds ratios (OR) with 95% confidence intervals (CI). All analyses were conducted in Python (pandas, statsmodels, SciPy).

In addition, a post hoc McNemar power analysis was performed for expert vs AI Top-1 comparisons using the observed discordant counts. This yielded 99.97% power for expert vs ChatGPT-5 (*b* = 31, *c* = 4) and 90.6% power for expert vs ChatGPT-4o (*b* = 29, *c* = 9) at *α* = 0.05, confirming that the study was adequately powered for these primary comparisons.

## Results

**Fig. 1:**
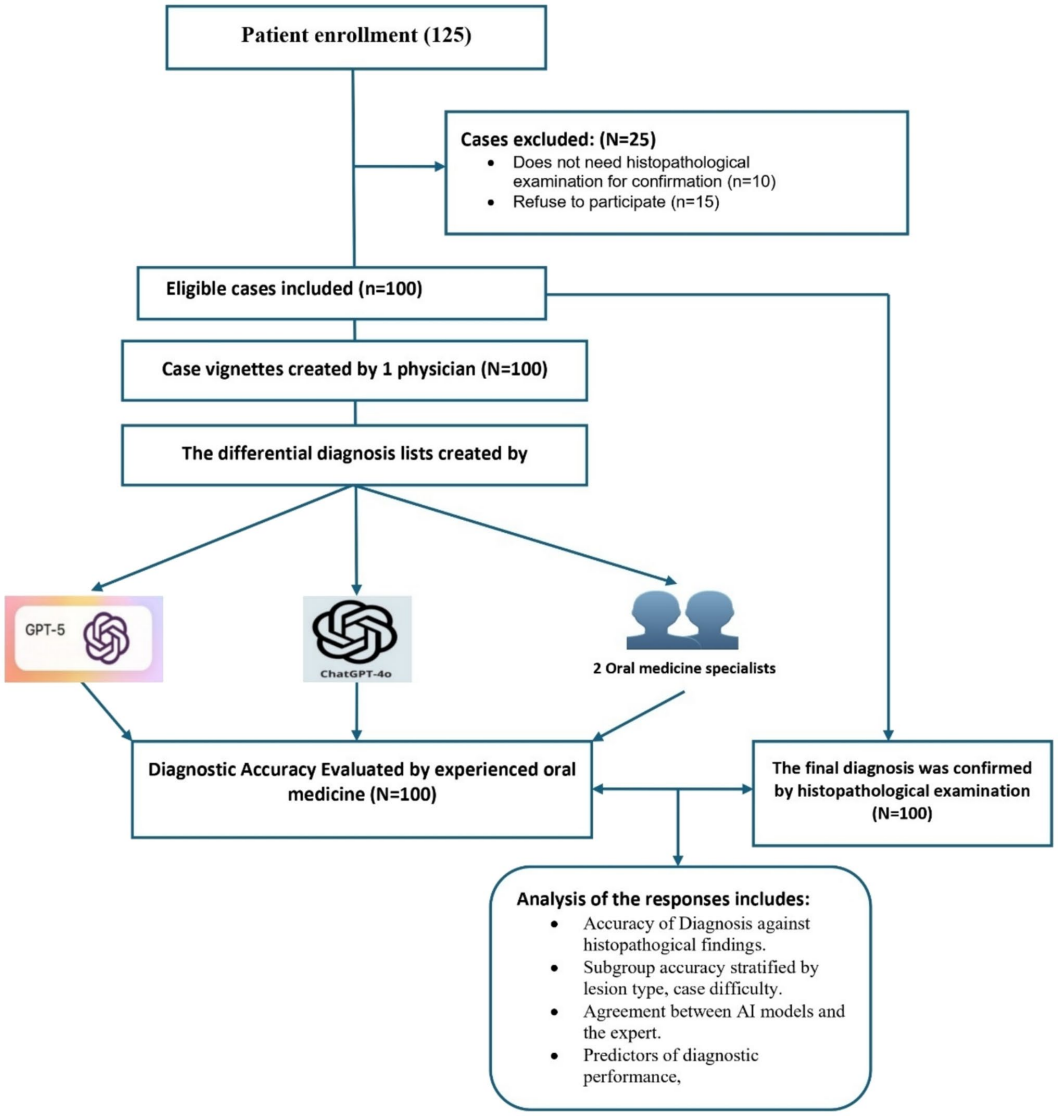
Study design

A total of 100 biopsy-confirmed oral lesion cases were analyzed after excluding 25 patients who either declined participation or did not require histopathological confirmation. Diagnostic accuracy was compared between ChatGPT-5, ChatGPT-4o, and the oral medicine expert. As illustrated in Fig. 1, ChatGPT-5 was presented first in all analyses, followed by ChatGPT-4o and the expert, with performance evaluated across Top-1, Top-3, and Top-5 ranked differential diagnoses.

### Comparison to expert diagnoses

The diagnostic accuracy of the two AI models and the expert consensus for oral lesion diagnosis is summarized in Table 1.

**Table 1:** Diagnostic accuracy of two LLM models and a human expert in oral lesions diagnosis at Top-1, Top-3, and Top-5 ranks, with pairwise McNemar’s tests and Cochran’s Q *p*-values

Comparison	ChatGPT-5 *n*(%)	ChatGPT-4o *n* (%)	Expert *n* (%)	Cochran’s Q *p*-value
Top-1	52 (52.0%)ᵃ	59 (59.0%) ᵃ	79 (79.0%)ᵇ	0.0000*
Top-3	72 (72.0%)ᵃ	77 (77.0%) ᵃ	88 (88.0%)ᵃ	0.0027*
Top-5	78 (78.0%)ᵃ	83 (83.0%) ᵃ	91 (91.0%)ᵃ	0.0117*

Values sharing a letter are not significantly different; values with no letter in common differ significantly. At Top-3 and Top-5, Cochran’s Q identified an overall difference across raters, but pairwise comparisons were nonsignificant after adjustment, so all raters share the same superscript

Cochran’s Q *p*-value tests the overall difference across raters; * indicates *p* < 0.05. All analyses use a paired design

Superscripts ᵃ,ᵇ indicate homogeneous subsets from Holm-adjusted pairwise McNemar’s exact tests (*α* = 0.05)

At the **Top-1** rank, correct diagnoses were achieved in 52.0% of cases by ChatGPT-5, 59.0% by ChatGPT-4o, and 79.0% by the expert consensus, which is consistent with or exceeds typical diagnostic accuracy rates reported in the literature for oral medicine experts [24, 25].

Pairwise McNemar’s exact tests with Holm adjustment revealed that the expert significantly outperformed both ChatGPT-5 (*p* = 0.0003) and ChatGPT-4o (*p* = 0.0041), whereas no significant difference was observed between ChatGPT-5 and ChatGPT-4o (*p* = 0.317). Cochran’s Q test confirmed a significant overall difference among raters (*p* < 0.0001). Effect size analyses indicated that the expert was about eight times more likely than ChatGPT-5 to be correct on discordant cases (OR = 7.75, 95% CI 2.74–21.95), corresponding to a 27% absolute accuracy difference (95% CI: 15.4%–38.6%). Against ChatGPT-4o, the expert remained significantly superior (OR = 3.22, 95% CI 1.53–6.81), with a 20% absolute difference (95% CI 7.9%–32.1%). Post hoc McNemar power was 99.97% for expert vs ChatGPT-5 and 90.6% for expert vs ChatGPT-4o, confirming the adequacy of the sample for these comparisons.

At the **Top-3** rank, the proportion of correct diagnoses increased to 72.0% for ChatGPT-5, 77.0% for ChatGPT-4o, and 88.0% for the expert. Pairwise comparisons showed no significant differences between any rater pairs (ChatGPT-5 vs ChatGPT-4o, *p* = 0.143; ChatGPT-5 vs expert, *p* = 0.056; ChatGPT-4o vs expert, *p* = 0.115), although the overall Cochran’s Q test remained significant (*p* = 0.0027).

**Fig. 2:**
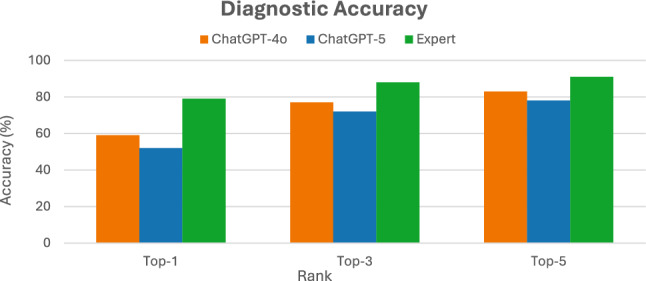
Bar chart displaying Top-1, Top-3, and Top-5 diagnostic accuracy (%) for ChatGPT-5, ChatGPT-4o, and human expert

At the **Top-5** rank, accuracies further improved to 78.0%, 83.0%, and 91.0% for ChatGPT-5, ChatGPT-4o, and the expert, respectively. No significant differences were found between ChatGPT-5 and ChatGPT-4o (*p* = 0.221), ChatGPT-5 and expert (*p* = 0.077), or ChatGPT-4o and expert (*p* = 0.118). However, the Cochran’s Q test still identified a significant overall difference among raters (*p* = 0.0117). (Fig. 2).

### Subgroup analysis by case characteristics

#### Diagnostic accuracy by lesion types

Table 2 summarizes the accuracy by lesion type and rank for ChatGPT-5, ChatGPT-4o, and the expert consensus. At the **Top-1** rank, the expert achieved the highest accuracy for inflammatory lesions (97.1%), significantly exceeding ChatGPT-5 (64.7%, *p* = 0.0003) and ChatGPT-4o (64.7%, *p* = 0.0003), while the AI models did not differ (*p* = 1.000). In benign lesions, the expert reached 72.7%, compared with 36.4% (ChatGPT-5, *p* = 0.125) and 27.3% (ChatGPT-4o, *p* = 0.063); AI vs AI yielded *p* = 0.500. Reactive (54.3–74.3%) and malignant lesions (35.0–60.0%) showed no statistically significant pairwise differences (all *p* > 0.05). Cochran’s Q indicated significant overall differences for inflammatory (*p* = 0.0005) and benign (*p* = 0.0302) lesions.

**Table 2:** Diagnostic accuracy of two LLM models and a human expert across oral lesion types and ranks, with exact McNemar pairwise comparisons and Cochran’s Q tests

Rank	Lesion (*n*)	ChatGPT-5	ChatGPT-4o	Expert	Cochran’s Q *p*-value
Top-1	Inflammatory (*n* = 34)	22 (64.7%)ᵃ	22 (64.7%)ᵃ	33 (97.1%)ᵇ	0.0005*
	Benign (*n* = 11)	4 (36.4%)ᵃ	3 (27.3%)ᵃ	8 (72.7%)ᵃ	0.0302*
	Reactive (*n* = 35)	19 (54.3%)ᵃ	22 (62.9%)ᵃ	26 (74.3%)ᵃ	0.1572
	Malignant (*n* = 20)	7 (35.0%)ᵃ	12 (60.0%)ᵃ	12 (60.0%)ᵃ	0.0622
Top-3	Inflammatory (*n* = 34)	29 (85.3%)ᵃ	30 (88.2%)ᵃ	34 (100.0%)ᵃ	0.0498*
	Benign (*n* = 11)	6 (54.5%)ᵃ	6 (54.5%)ᵃ	9 (81.8%)ᵃ	0.1054
	Reactive (*n* = 35)	25 (71.4%)ᵃ	26 (74.3%)ᵃ	30 (85.7%)ᵃ	0.1988
	Malignant (*n* = 20)	12 (60.0%)ᵃ	15 (75.0%)ᵃ	15 (75.0%)ᵃ	0.4066
Top-5	Inflammatory (*n* = 34)	30 (88.2%)ᵃ	32 (94.1%)ᵃ	34 (100.0%)ᵃ	0.1353
	Benign (*n* = 11)	6 (54.5%)ᵃ	7 (63.6%)ᵃ	9 (81.8%)ᵃ	0.1738
	Reactive (*n* = 35)	26 (74.3%)ᵃ	28 (80.0%)ᵃ	32 (91.4%)ᵃ	0.0784
	Malignant (*n* = 20)	16 (80.0%)ᵃ	16 (80.0%)ᵃ	16 (80.0%)ᵃ	1.0000

Superscripts denote results of pairwise McNemar’s exact tests with Holm adjustment (*α* = 0.05). Values sharing the same letter are not significantly different; values with no letter in common differ significantly. When all values share the same superscript, this indicates that no pairwise comparison was statistically significant, even if the overall Cochran’s Q test reached significance

At the **Top-3** rank, performance improved across all lesion types. For inflammatory lesions, accuracy was 100% for the expert, 88.2% for ChatGPT-4o (*p* = 0.500 vs expert), and 85.3% for ChatGPT-5 (*p* = 0.250 vs expert; AI vs AI *p* = 1.000). Benign lesions (54.5–81.8%), reactive lesions (71.4–85.7%), and malignant lesions (60.0–75.0%) showed no significant differences in any pairwise comparison (all *p* ≥ 0.125). Only inflammatory lesions had a significant Cochran’s Q (*p* = 0.0498).

**Fig. 3:**
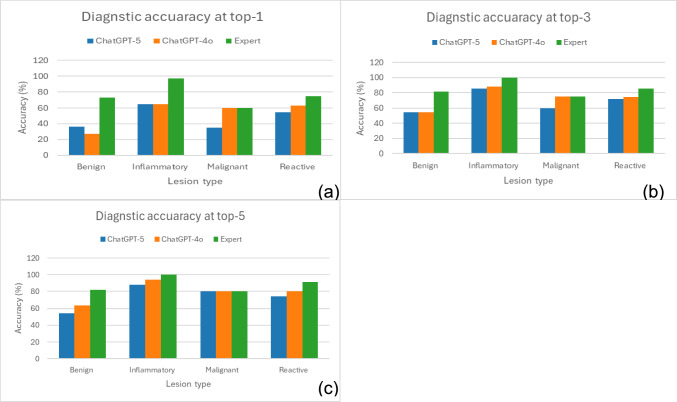
Subgroup diagnostic accuracy by lesion type for ChatGPT-5, ChatGPT-4o, and the expert consensus. **a** Top-1 rank accuracy, **b** Top-3 rank accuracy, and **c** Top-5 rank accuracy. Accuracy is presented as the percentage of biopsy-confirmed oral lesion cases correctly identified within each lesion category (benign, inflammatory, malignant, and reactive)

At the **Top-5** rank, accuracies reached ≥ 88.2% for inflammatory and ≥ 80.0% for malignant lesions. For inflammatory lesions, the expert (100%) was not significantly different from ChatGPT-4o (94.1%, *p* = 0.500) or ChatGPT-5 (88.2%, *p* = 0.125; AI vs AI *p* = 0.500). Benign (54.5–81.8%) and reactive lesions (74.3–91.4%) again showed no significant pairwise differences (all *p* ≥ 0.063), and malignant lesions had identical accuracy (80.0%) across all raters (*p* = 1.000). Cochran’s Q results were nonsignificant for all lesion types at this rank (all *p* ≥ 0.0784). Overall accuracy trends are visually summarized in Fig. 3**.**

#### Diagnostic accuracy by case difficulty

Table 3 presents the diagnostic accuracy stratified by case difficulty level. At the **Top-1** rank, the expert achieved the highest accuracy for middle-difficulty cases (87.5%), followed by ChatGPT-4o (67.5%, *p* = 0.063 vs expert) and ChatGPT-5 (65.0%, *p* = 0.031 vs expert), with no significant difference between AI models (*p* = 0.500). In high-difficulty cases, accuracy ranged from 45.7% (ChatGPT-5) to 68.6% (expert), but pairwise differences were not significant (all *p* ≥ 0.063). For low-difficulty cases, the expert achieved 80.0% accuracy, significantly higher than ChatGPT-5 (40.0%, *p* = 0.004) and ChatGPT-4o (48.0%, *p* = 0.031), while the AI models did not differ (*p* = 0.500). Cochran’s Q indicated significant overall differences for middle- (*p* = 0.0173) and low- (*p* = 0.0052) difficulty cases.

**Table 3:** Diagnostic accuracy of two LLM models and a human expert across difficulty levels and ranks, with exact McNemar pairwise comparisons and Cochran’s Q tests

Rank	Difficulty (*n*)	ChatGPT-5	ChatGPT-4o	Expert	Cochran’s Q *p*-value
Top-1	Middle (*n* = 40)	26 (65.0%)ᵃ	27 (67.5%)ᵃ	35 (87.5%)ᵃ	0.0173*
	High (*n* = 35)	16 (45.7%)ᵃ	20 (57.1%)ᵃ	24 (68.6%)ᵃ	0.0594
	Low (*n* = 25)	10 (40.0%)ᵃ	12 (48.0%)ᵃ	20 (80.0%)ᵃ	0.0052*
Top-3	Middle (*n* = 40)	31 (77.5%)ᵃ	34 (85.0%)ᵃ	38 (95.0%)ᵃ	0.0098*
	High (*n* = 35)	24 (68.6%)ᵃ	25 (71.4%)ᵃ	28 (80.0%)ᵃ	0.4437
	Low (*n* = 25)	17 (68.0%)ᵃ	18 (72.0%)ᵃ	22 (88.0%)ᵃ	0.1225
Top-5	Middle (*n* = 40)	33 (82.5%)ᵃ	37 (92.5%)ᵃ	38 (95.0%)ᵃ	0.0150*
	High (*n* = 35)	28 (80.0%)ᵃ	27 (77.1%)ᵃ	29 (82.9%)ᵃ	0.8071
	Low (*n* = 25)	17 (68.0%)ᵃ	19 (76.0%)ᵃ	24 (96.0%)ᵃ	0.0202*

Superscripts denote results of pairwise McNemar’s exact tests with Holm adjustment (*α* = 0.05). Values sharing the same letter are not significantly different; values with no letter in common differ significantly. When all values share the same superscript, this indicates that no pairwise comparison was statistically significant, even if the overall Cochran’s Q test reached significance

At the **Top-3** rank, performance improved for all difficulty levels. In middle-difficulty cases, the expert achieved 95.0% accuracy, significantly higher than ChatGPT-5 (77.5%, *p* = 0.016), but not significantly different from ChatGPT-4o (85.0%, *p* = 0.125); AI models did not differ (*p* = 0.500). High-difficulty (68.6–80.0%) and low-difficulty cases (68.0–88.0%) showed no statistically significant pairwise differences (all *p* ≥ 0.063). Cochran’s Q was significant only for middle-difficulty cases (*p* = 0.0098).

At the **Top-5** rank, accuracies were highest overall, with middle-difficulty cases reaching 95.0% for the expert, 92.5% for ChatGPT-4o (*p* = 0.500 vs expert), and 82.5% for ChatGPT-5 (*p* = 0.031 vs expert; AI vs AI *p* = 0.125). In high-difficulty cases, accuracies were closely matched (77.1–82.9%) without significant differences. For low-difficulty cases, the expert (96.0%) significantly outperformed ChatGPT-5 (68.0%, *p* = 0.004), but not ChatGPT-4o (76.0%, *p* = 0.063); AI models did not differ (*p* = 0.500). Cochran’s Q identified significant differences for middle- (*p* = 0.0150) and low- (*p* = 0.0202) difficulty cases.

### Agreement of LLM with human expert

Agreement analysis between each AI model and the expert rater demonstrated increasing concordance at broader rank thresholds. For ChatGPT-5, percent agreement rose from 65.0% at Top-1 to 79.0% at Top-5. Corresponding Cohen’s κ values declined slightly (0.283 to 0.223), remaining in the *fair* range, whereas AC1 increased from 0.361 (fair) to 0.715 (substantial). Similarly, ChatGPT-4o achieved 62.0% agreement at Top-1, rising to 82.0% at Top-5, with κ values between 0.151 and 0.215 (fair) and AC1 values improving from 0.336 (fair) to 0.767 (substantial). In both models, AC1 consistently exceeded κ, reflecting its greater robustness to prevalence and bias. Overall, both LLMs showed progressively stronger alignment with expert diagnoses as the rank threshold expanded. While numerical differences in agreement metrics were observed between ChatGPT-5 and ChatGPT-4o, no statistically significant difference in diagnostic accuracy was found between the two models at any rank (Table 4**).**

**Table 4:** Agreement with expert rater (*n* = 300): percent agreement, Cohen’s κ, and AC1

Model	Rank	Percent agreement	Cohen’s κ	AC1
ChatGPT-5	Top-1	65.0	0.283	0.361
	Top-3	74.0	0.219	0.618
	Top-5	79.0	0.223	0.715
ChatGPT-4o	Top-1	62.0	0.151	0.336
	Top-3	77.0	0.220	0.677
	Top-5	82.0	0.215	0.767

AC1 was computed for a two-rater, binary outcome (correct/incorrect) using chance agreement based on the mean positive rate of the two raters; values are rounded to three decimals. Higher AC1 indicates stronger agreement beyond chance (approximate interpretation: 0.41–0.60 = moderate, 0.61–0.80 = substantial)

## Regression analysis of diagnostic predictors

Multivariable logistic regression analysis (Table 5) demonstrated that lesion type was the main predictor of diagnostic accuracy for both ChatGPT-5 and ChatGPT-4o across ranks, while difficulty level showed no significant association. For ChatGPT-5, inflammatory lesions were significantly more likely to be correctly diagnosed at **Top-5** (OR = 6.48, 95% CI 1.01–41.64, p = 0.049), but no significant associations were observed at Top-1 or Top-3. For ChatGPT-4o, inflammatory lesions were strongly associated with higher accuracy at **Top-3** (OR = 9.87, 95% CI 1.60–60.85, p = 0.014) and **Top-5** (OR = 10.56, 95% CI 1.49–74.81, p = 0.018). Reactive and malignant lesions showed positive, but nonsignificant odds ratios in most ranks. Difficulty level (low or middle vs. high) was not a statistically significant predictor in any model. These findings suggest that lesion category, particularly inflammatory lesions, exerts a stronger influence on AI diagnostic accuracy than case difficulty, and that performance advantages become more apparent at broader ranks.

**Table 5:** Multivariable logistic regression predicting diagnostic accuracy for ChatGPT-5 and ChatGPT-4o across Top-1, Top-3, and Top-5 ranks

Rank	Predictor	ChatGPT-5			ChatGPT-4o		
		OR	95% CI	*p*-value	OR	95% CI	*p*-value
Top-1	Lesion: inflammatory	1.85	0.35–9.80	0.470	3.04	0.58–15.83	0.187
	Lesion: reactive	1.73	0.41–7.30	0.457	2.60	0.61–11.06	0.197
	Lesion: malignant	0.63	0.09–4.63	0.648	2.91	0.39–21.97	0.298
	Difficulty: low	0.53	0.11–2.54	0.429	0.65	0.14–3.02	0.579
	Difficulty: middle	1.24	0.36–4.28	0.733	1.51	0.44–5.21	0.507
Top-3	Lesion: inflammatory	5.63	0.94–33.67	0.058	**9.87**	**1.60–60.85**	0.014*
	Lesion: reactive	2.20	0.52–9.28	0.283	2.74	0.63–11.88	0.179
	Lesion: malignant	1.44	0.17–11.82	0.735	2.63	0.31–22.51	0.379
	Difficulty: low	1.22	0.21–7.17	0.827	1.26	0.22–7.27	0.794
	Difficulty: middle	0.98	0.21–4.54	0.981	1.10	0.24–5.10	0.901
Top-5	Lesion: inflammatory	**6.48**	**1.01–41.64**	**0.049**	**10.56**	**1.49–74.81**	0.018*
	Lesion: reactive	2.37	0.56–10.07	0.243	3.10	0.63–15.22	0.162
	Lesion: malignant	4.26	0.46–39.26	0.201	3.13	0.32–30.37	0.322
	Difficulty: low	1.29	0.21–7.86	0.780	1.89	0.29–12.33	0.507
	Difficulty: middle	1.38	0.28–6.76	0.690	3.44	0.65–18.10	0.144

Odds ratios (OR) are adjusted for lesion type and difficulty level. Reference categories are benign for lesion type and high for difficulty level. OR > 1 indicates higher odds of a correct diagnosis compared with the reference category. Models were fit separately for each AI model and rank. Boldface indicates statistical significance (*p* < 0.05). Wide confidence intervals in some strata reflect small sample sizes

## Discussion

Using a paired design that enables direct comparison with skilled clinicians, this study is the first prospective, biopsy-confirmed assessment of ChatGPT-5 and ChatGPT-4o in the diagnosis of oral lesions. Our design provides better internal validity and clinically relevant benchmarks than previous analyses, which relied on retrospective case vignettes.

Across all diagnostic ranks, our study reveals a pronounced performance difference between AI models (ChatGPT-5 and ChatGPT-4) and a human expert. The expert’s 79% accuracy at the Top-1 rank beats both AI models (~ 52–59%), but at Top-3 and Top-5, the gaps close and the statistical significance decreases. Inflammatory and benign lesions exhibit more noticeable differences, especially at the strictest (Top-1) ranking, according to subgroup analyses. While case difficulty does not seem to affect model performance, logistic regression identifies lesion type, particularly inflammatory lesions, as a significant positive predictor of AI accuracy at broader rank thresholds (Top-3 and Top-5).

The better performance of both AI models as observed on inflammatory lesions, particularly at broader diagnostic ranks, is consistent with the hypothesis that such conditions should be easier for general-purpose LLMs to learn. There are several factors that may account for this. Firstly, inflammatory lesions (e.g., lichen planus and aphthous ulcers) often have well-documented, classic clinical presentations that are well described extensively in the medical literature and thus likely well represented in the models’ training data [32, 33]. Secondly, since they are relatively prevalent in general and in dental practice, inflammatory conditions are probably overrepresented in text and image corpora, i.e., they constitute a denser data foundation for pattern recognition [34]. Finally, unlike the extensive morphological heterogeneity and subtle atypia of the majority of malignant or premalignant lesions, inflammatory diseases present with less variation in presentation and are therefore less challenging for AI classification [11, 25]. While these explanations are speculative, they raise testable hypotheses that future work could resolve through systematic training data distribution audits and model attention mechanism inspection.

Our results are consistent with broader trends in the literature, which indicate that while AI approaches have promise, they are still surpassed by human expertise in specific, high-risk diagnoses. For instance, research on deep learning for the classification of oral lesions shows high sensitivity and specificity in the best circumstances, but it hardly ever outperforms human clinicians in terms of Top-1 accuracy [35, 36]. According to a recent narrative review, AI performance varies widely, with accuracy ranging from about 63 to 100%, sensitivity from 70 to 100%, and specificity from 57 to 100%. This shows promise, but also variability and the need for careful interpretation [34].

While AI systems that use radiographic imaging have demonstrated significant speed and agreement with human clinicians, they still fall short of expert diagnostic consistency regularly [35, 37]. Likewise, when used to diagnose oral mucosal images, ChatGPT-4o demonstrated high sensitivity and specificity and did not substantially deviate from clinician performance again, in a specific context [36].

We appreciate that radiographic AI systems are based on structured imaging data, which inherently differs from the narrative case data used here, and are therefore not comparable. Our intent in citing imaging studies is to provide context for AI performance trends across diagnostic modalities, and not to imply comparability.

Our findings are consistent with previous imaging-based AI studies that showed high sensitivity in identifying cancers and other oral premalignant lesions [16, 29]. ChatGPT-5, in contrast to image-only systems, incorporates contextual case data and narrative, which makes it especially helpful in situations requiring multimodal diagnostic reasoning. These methods collectively point to a hybrid future where imaging AI and LLMs work in tandem to improve diagnostic precision and speed referrals.

Our research showed that ChatGPT-5 closely resembles expert clinician performance in producing differential diagnoses for oral lesions, achieving competitive accuracy, especially at the Top-3 rank. These findings align with broader trends showing that LLMs can approach or occasionally match human diagnostic reasoning in controlled settings. For instance, prior work in oral medicine demonstrated that ChatGPT (and Copilot) could generate accurate differentials from text-based clinical scenarios, albeit without histopathology confirmation, and with scoring systems paralleling ours [23]. This situates ChatGPT-5 as a support tool that complements clinical expertise rather than replacing it.

Our results add to the body of evidence showing AI, and more recently LLMs, can help with differential diagnosis and triage for oral diseases. The usefulness of AI in frontline screening and decision support is highlighted by meta-analytic data that demonstrate strong AI performance for identifying oral cancer and potentially malignant disorders (OPMDs) on clinical imaging [16]. The current findings, which use rank-based scoring akin to our framework, are consistent with prospective comparisons in oral medicine that demonstrate that LLMs can approximate consultants on text-based clinical scenarios [23].

Similar to our subgroup effects and difficulty strata, recent cross-sectional studies that benchmark ChatGPT variants in oral pathologies show variability across prompts and conditions, while also reporting clinically usable accuracy [15, 20, 38]. When combined, these reports establish next-generation LLMs as reliable tools for making preliminary differential diagnoses in oral lesions, particularly when applied under the supervision of an expert.

Our findings expand on earlier analyses of previous iterations of the model. ChatGPT-4’s accuracy in neuro-ophthalmology case reports was 82%, which is close to the 86% accuracy of clinicians, while ChatGPT-3.5’s accuracy was only 59% [39]. Although reliability issues still exist, other clinical tests of GPT-4 have also demonstrated better performance than GPT-3.5, including in medical examination contexts [38]. Therefore, the improved performance of ChatGPT-5 in our investigation aligns with a noted increase in LLM capabilities.

According to recent studies, combining the outputs of several LLMs increases diagnostic accuracy more than using just one model. When synthesizing responses from multiple LLMs, one study spanning 200 real-life clinical vignettes reported an average accuracy of approximately 75%, while individual models showed an accuracy of approximately 59% [40].

Other studies have shown that combining the output of several LLMs minimizes the biases and errors of individual models to produce more accurate and calibrated medical reasoning [41, 42]. In addition, ensemble methods are some of the best approaches to maximize the efficacy of AI-based diagnostic models, as established through systematic reviews [43, 44].

This implies that, in addition to the already excellent performance we observed, future research may further improve accuracy and reliability by combining ChatGPT-5 with other LLM outputs or aggregating various prompts.

Our results imply that ChatGPT-5 may be used as a first-line support tool in oral medicine clinics from a translational standpoint. The model may help prioritize urgent biopsy referrals, expedite triage, and reduce diagnostic delays that have a direct impact on survival outcomes by reliably classifying malignant and potentially malignant disorders within its Top-3/Top-5 differentials. ChatGPT-5, in contrast to image-only systems, incorporates contextual case data and narrative, which makes it especially helpful in situations requiring multimodal diagnostic reasoning. These methods collectively point to a hybrid future where imaging AI and LLMs complement each other to improve diagnostic precision and speed referrals.

### Implications and interpretations

In clinical practice, differential diagnosis usually spans two to five conditions rather than a single guess, guiding decisions on biopsy or referral. Thus, the Top-3 and Top-5 frameworks mirror real-world diagnostic reasoning and provide a safety margin, ensuring malignant or potentially malignant lesions are not overlooked even if Top-1 precision is imperfect.

*Top-1 performance gap*: The continuously lower Top-1 accuracy of AI models shows that, despite their ability to generate reliable differential diagnoses, they still lack the precision and complex reasoning that come from clinical training, experience, and thorough patient integration. They may struggle to identify subtle presentation cues or unusual lesions.

*Rank-dependent improvements*: Even though AI may not immediately recognize the correct diagnosis, it consistently counts it among the plausible possibilities, as evidenced by the convergence of AI performance with expert levels at Top-3 and Top-5. This illustrates how AI could be used as a diagnostic tool that aids physicians in making decisions rather than taking their place.

Our Top-3 and Top-5 analyses’ practical significance originates from their compatibility with clinical workflows. For example, current oral cancer referral guidelines usually call for an urgent biopsy or referral if a malignant lesion is found within the Top-3 differentials recommended by ChatGPT-5. In a similar vein, adding potentially malignant disorders (PMDs) to the Top-5 guarantees that these cases are not missed during triage, promoting safe patient care.

We particularly emphasize that the Top-3 and Top-5 are not unjustified extensions but rather chosen deliberately to represent the variety of conditions typically considered by doctors during real diagnostic reasoning. Compared to an ideal but clinically useless Top-100 system, these targets assure gains in performance in terms of significant help for safe and actionable patient care.

From a patient management perspective, inclusion of malignant or premalignant conditions in the Top-3/5 differential allows clinicians to take immediate action through biopsy or referral suggestions without delay, so diagnostic delay is minimized. In high-volume centers, this might reduce cognitive burden for clinicians, speed up triage, and provide insurance against cancer misses. Thus, even if the precision of Top-1 is suboptimal, Top-3/5 performance translates to actual gains in safety and efficiency of care.

*Lesion type dependency*: AI’s superior performance on inflammatory lesions, especially at broader ranks, is consistent with models trained on more common, well-represented patterns. Inflammatory lesions are likely easier for LLMs to comprehend because they share more classic features and may have richer representation in training data. Benign, reactive, or malignant lesions may reduce the generalizability of the model due to their greater variety.

Since ChatGPT-5’s Top-3 accuracy allows for effective triage and differential narrowing, it could be used as a supportive tool in oral medicine, especially in high-volume settings. A hybrid workflow of AI-assisted screening with clinician oversight may improve diagnostic speed and safety by lowering the cognitive load on clinicians and identifying cases that are unclear for expert review.

*Case difficulty*: Since there are no observable relationships between difficulty levels and AI performance, it would seem that complexity is not the only factor contributing to model errors. Instead, systematic biases, like an imbalance in the dataset or a lack of clinical context, may be the source of inaccuracies.

### Conformity to more comprehensive evidence

A study that employed ChatGPT-4 with a “Chain-of-Thought” prompting method (CoT) to enhance reasoning and response quality for oral and maxillofacial queries showed that prompting techniques can enhance AI interpretability and performance [45]. This suggests that enhancing AI input methods could boost clinical utility, which is a worthwhile endeavor to reduce the Top-1 gap.

### Strengths of the study

One of the key advantages of this study is its paired design, which allowed for direct within-case comparisons between AI models and a human expert while controlling for inter-case variability. Strong statistical methods, like McNemar’s exact test with Holm adjustment and Cochran’s Q test, ensure that observed differences are both statistically sound and clinically significant. The stratification of analyses by lesion type and case difficulty also provided a nuanced understanding of where AI models perform well and where they fall short, as it provided granular insight instead of aggregated averages.

The evaluation of concordance between expert diagnoses and AI predictions was strengthened by applying both Cohen’s κ and AC1. While κ is widely used, it is sensitive to prevalence and marginal distributions; AC1 provides a more robust adjustment for chance agreement and prevalence bias. Using both measures enhances the internal validity and interpretability of the results.

### Limitations

The study has some limitations that need to be considered in the interpretation of results. First, subgroup analyses of certain lesion types (particularly benign and malignant) were limited by low numbers with broad confidence intervals and reduced statistical resources to detect differences across these categories. Second, the general-purpose design of tested AI models may limit their specificity to unusual or highly specific oral conditions, and thus emphasize the potential for future, domain-specific fine-tuning.

A central point to consider is that our study design, employing biopsy-proven cases to yield a high-quality histopathological gold standard, by design, focuses on the differential generation stage rather than on primary clinical triage. Even though this maximizes internal validity, it means that we cannot examine the potentially most valuable possible use of AI: influencing the clinician’s first decision to biopsy, monitor, or reassure at all. Consequently, our findings demonstrate the models’ capacity to rank diagnoses for a known lesion, but do not assess their utility in preventing underscreening or guiding management decisions in a larger clinical population.

Further, the clinical vignettes, though standardized, were not exhaustive and did not include all of the potential descriptors a clinician might provide (e.g., history of recurrence, careful lesion duration, or adjunctive imaging features), which can affect the generalizability of the findings. The use of two experts’ consensus, although stringent, may not represent the larger population of oral medicine practitioners.

Importantly**,** although OSCC comprised the largest malignant entity in our dataset due to its prevalence and prognostic importance, it must be acknowledged that OSCC is rarely a source of diagnostic error for specialist oral pathologists. Its clinical features may overlap with other oral lesions for dental clinicians, but greater diagnostic challenges in pathology typically arise with malignant lymphomas, osteosarcomas, and hematologic-related soft tissue tumors. Future validation across such diagnostically complex entities will therefore be essential.

Finally**,** from a clinical safety perspective, most noteworthy is that the value of the Top-3 and Top-5 frameworks serve as a bulwark against blind spots. One important finding is that neither the AI model nor the expert entirely missed any malignant lesions at the Top-5 level, which means these tools may be a high-recall measure of safety to not miss something significant. However, the impressive expert differentiation at Top-1 underscores that the final, authoritative diagnosis and related management decisions must remain within the clinician’s prerogative. LLMs, on this premise, must be integrated as adjunctive triage tools in a supervised clinical pathway, but not as stand-alone diagnostic tools.

### Ethical challenges

The safe clinical adoption of LLMs depends on ethical and regulatory factors. Despite ChatGPT-5’s encouraging accuracy in producing ranked differentials, AI misdiagnosis, especially false negatives in malignant or potentially malignant lesions, could jeopardize patient safety and cause treatment delays. Additionally, general-purpose LLMs are not categorized as regulated medical devices by current frameworks; however, they would probably be subject to FDA (US) or CE (Europe) approval pathways once they are incorporated into diagnostic workflows. The necessity of cautious governance, openness in model training data, and clear clinician supervision before clinical deployment is highlighted by this regulatory ambiguity. Therefore, rather than serving as a stand-alone diagnostic system, our study supports ChatGPT-5’s function as an auxiliary decision support tool.

### Future directions

*Better collaboration between clinicians and AI specialists*: AI systems may present ranked differentials for clinicians to select from, potentially reducing diagnostic error.

Using structured reasoning prompts, which are effective for ChatGPT-4 in oral queries, prompt engineering, and CoT techniques [45], could increase specificity.

*Specialized training*: Models may be better at discriminating between reactive, malignant, and benign oral lesions if they are trained on carefully chosen datasets.

*Multimodal integration*: By incorporating histopathology, imaging (radiographs, photographs), and clinical narratives, AI models may be better able to replicate the expert’s comprehensive approach.

*Larger, multicenter validation*: Increasing the size of datasets across institutions could improve statistical power and generalizability.

*Patient-centered outcomes*: The effects of AI tools on patient education, communication, and collaborative decision-making should also be investigated in future studies. AI-generated explanations of differential diagnoses, for instance, could give patients more confidence in the diagnostic process, enable them to ask well-informed questions, and help them understand their condition better.

*Safety end points and workflow simulation*: Future prospective trials should explicitly track critical misses (malignant or PMDs excluded from the Top-5) as dedicated safety outcomes. In addition, workflow-based studies are needed to evaluate whether AI-assisted triage reduces diagnostic delays and improves time to biopsy or referral in real-world oral medicine settings.

## Conclusions

Our results support the complementary role of AI in oral medicine: ChatGPT-5/4o shows promising potential when given a wider answer space, even though it is not as accurate as expert analysts in pinpoint accuracy (Top-1). While difficulty seems to be less of a determining factor, lesion type, particularly inflammatory, has a significant impact on AI success. Future developments could close the gap and integrate AI as a supplement in clinical workflows through quick refinement, customized training, and multimodal models.

Our work offers some of the most thorough evidence to date on the diagnostic capabilities of LLMs in oral medicine by using a prospective, biopsy-validated, paired design, establishing ChatGPT-5 as a possible supplementary tool in workflows for differential diagnosis.

## Supplementary Information

Below is the link to the electronic supplementary material.

Supplementary file1 (DOCX 1353 KB)

## Data Availability

Research data supporting this publication is available from the corresponding author upon request.
