# Supplementary material for: ChatGPT-5 vs oral medicine experts for rank-based differential diagnosis of oral lesions: a prospective, biopsy-validated comparison
Source: Odontology. 2025 Nov 17;114(3):1743–56. doi: 10.1007/s10266-025-01242-x (PMC13319845; doi:10.1007/s10266-025-01242-x)
Supplement: Supplementary file 1 — Supplementary file1 (DOCX 1353 KB) [file 10266_2025_1242_MOESM1_ESM.docx]

**Section S1. Anchor Vignettes Used for Model Priming**

**Demographic data:**

- Age: 70 years
- Gender: Female
- Marital status: Married, with three adult children

**Medical history:**

- Type 2 diabetes mellitus, well controlled with oral hypoglycemics
- No other significant comorbidities
- No tobacco or alcohol use

**Chief complaint:**

- Severe burning sensation of the inner cheek mucosa bilaterally for the past 2 years, exacerbated by spicy or acidic foods, with episodes of remission and exacerbation

**Extraoral examination:**

- No extraoral or mucocutaneous lesions detected

**Intraoral findings:**

- **Right buccal mucosa (Figure a):** A well-demarcated erythematous atrophic area measuring approximately 2 × 3 cm, surrounded by white keratotic reticular striae with bluish-white radiating lines extending peripherally
- **Left buccal mucosa (Figure b):** Multiple keratotic reticular papules on red atrophic mucosa, accentuated on stretching; lesions were non-scrapable, with bluish-white radiating lines at the periphery.


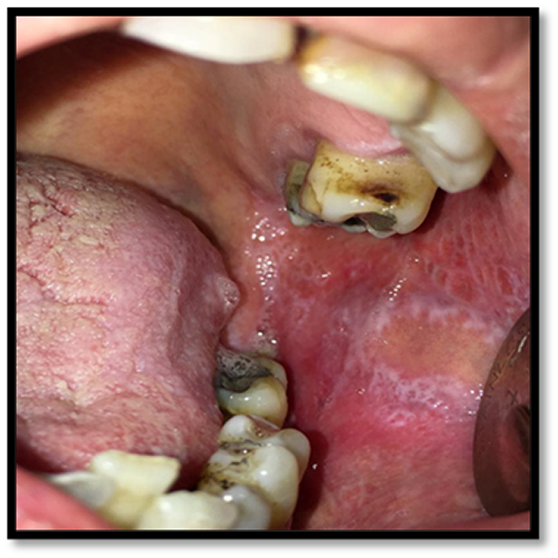

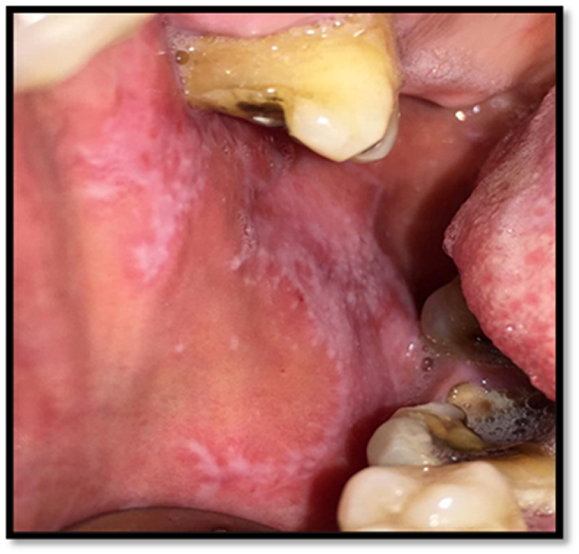


**Figure b**

**Figure a**
